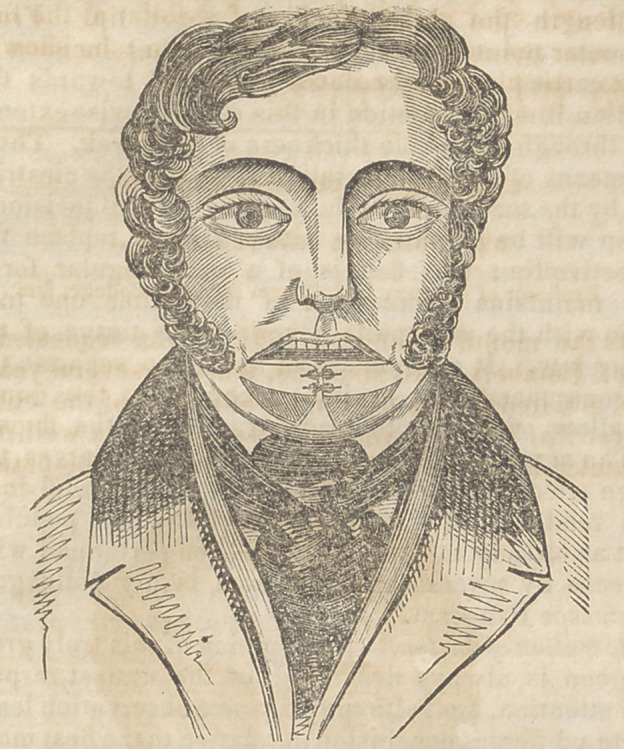# Cheiloplastic Operation

**Published:** 1844-07-13

**Authors:** Thomas D. Mütter

**Affiliations:** Professor of Surgery in Jefferson Medical College, &c.


### CHEILOPLASTIC OPERATION.

BY THOMAS D. MÜTTER, M. D.,

Professor of Surgery in Jefferson Medical College, &c.

In the month of January, 1842, I was requested to see I. Lambert, aged about 50, who, for several years, had suffered from a cancerous affection of the entire lower lip. The appearance of the disease is well represented in fig. 1. The general health of the patient being excellent, the glands in the vicinity apparently perfectly sound, and the season of the year favourable, I determined to attempt at once the removal of the disease, and at the same time restore the lip by a plastic operation. Accordingly, the patient was brought before my class on the regular clinical day, and the following operation performed.

Having seated him in a favourable position, with his head supported against the chest of an assistant, I proceeded to the removal of the entire diseased mass, by a semi-elliptical incision, which started from the commissure of the mouth on one side, and terminated at a corresponding point on the other. (See diagram, curved line A B.) From the centre of this line two slightly curved incisions, indicated by the lines C D and C E, were carried downwards and outwards, until they reached the base of the inferior maxillary bone.

It is obvious that these incisions were separated from each by a triangular piece of skin, the superior angle of which nearly reached the first incision, A B. Then, from the terminal extremities of the incisions C D, and C E, two others were carried upwards along the base of the lower jaw, until they reached a point opposite the initial and terminal points of the incision A B. Two quadrangular flaps, A C E F, and B C D G, were thus marked out, and immediately detached from the subjacent bone.

The hemorrhage having been arrested, and the patient allowed a few minutes of repose, the flaps were raised and placed in the position originally occupied by the lower lip, and then united to each other at the mesial line, and also by their lower thirds to the triangular piece of integument, by means of the twisted suture. By the elevation of these flaps, a raw surface on each side was left to heal by the modelling process, or by granulation. The parts were dressed with the "tepid water dressing," the patient placed in bed, with his head elevated, and a rigid antiphlogistic system of treatment ordered. Nothing of interest in the subsequent management of the case presented itself; the parts healed kindly, and the patient recovered, without a trace of the disease remaining. More than two years have elapsed since the performance of the operation, and Mr. Lambert is perfectly well, and actively engaged in business.

*Remarks.*

From the conspicuousness of the organ, its utility in articulation, and also in the prevention of an involuntary and incessant flow of saliva, the lower lip may be considered one of the most important portions of the face. Unfortunately, it is exceedingly prone to diseases of various kinds, especially tumours and ulcers, requiring, for their relief, the loss of either a portion or the whole of the organ involved. It would appear that its importance was long since recognized, and attempts made by the older surgeons to remedy its loss. But it is to our modern brethren, especially Dieffenbach, Liston, Velpeau, Roux, Lisfranc, Dupuytren, Blandin, Blasius, Zies, and Rigaud, that we are indebted for the most valuable information relative to the best modes of curing its diseases, or remedying its destruction.

Velpeau classes all the operations for the restoration of a lip under three general methods: the "*Italian*," "*Indian*" and *French*—each one of which comprises a vast number of "special methods," the result of the ingenuity of the operator, and the exigencies of the case. To these general methods, might be added that which Graëfe has designated as the "*German*." Taliacotius, and most of the older surgeons, resorted to the "*Italian*" plan of procedure; while the moderns, almost to a man, prefer some modification of either the *Indian*, *French*, or *German*. It would be worse than useless to enter into a description of all the operations that have been devised, but a brief sketch of the most novel and important may prove both useful and interesting to those not familiar with this department of plastic surgery.

*Chopart's Operation.*—The operation practised by Chopart consisted in making on each side of the diseased tissue, a perpendicular incision, which extended from the margin of the lip to a point below the base of the lower jaw. Disecting up the flap inclosed between the incisions, he carefully removed from its upper margin all the affected tissue, either by a *transverse* or *curvilinear* cut. Then, pulling upon what remained of the flap, he brought its upper edge to the level of the margin of the natural lip, and there retained it by suture, straps, and placing the head of the patient in such a position as to prevent all strain upon the part.

This method, though apparently simple and easy of execution, does not generally answer, in consequence of the subsequent contraction of the tissue. Nevertheless, it is well thought of by Velpeau, Rigaud, and some others. In my own cases, I have been obliged to perform a second operation, similar to that proposed by Thévenin, where the tissues are tight, and refuse to yield readily. viz: a *transverse incision* about an inch below the free margin of the lip. By doing this, and thus taking off all traction upon what forms the new lip, I have succeeded in making a very good cure.

*Horn, or Roonhuysen's Operation.*—When the tumour or ulcer to be removed is small, a common V-shaped incision, including the whole mass, is sufficient. The raw edges of the wound are brought together, and treated like a case of common hare-lip; but where the mass is large, this process is sure to diminish the orifice of the mouth, and thus give rise to deformity and inconvenience. To obviate this difficulty, it was proposed by Horn to detach the adjacent parts by free dissection from the maxillary bones, which would of course afford more material for the lip. The only objection to this method is the circumstance that, in many cases, the orifice of the mouth is rendered so small as to be almost useless, besides presenting great deformity.

*Operation of Dupuytren.*—This, in ordinary cases, was nothing more than cutting away by a semi-elliptical incision all the diseased tissue. Granulations spring up from the margin of the healthy skin, occupy in part the place of the original lip, and conceal to a certain extent the deformity. It is only in mild cases, however, that such a measure could succeed. In more desperate ones, Dupuytren himself resorted to some of the usual methods employed by others.

*Celsian Operation.*—Celsus, who was in truth one of the best plastic surgeons that ever lived, proposed, in cases where great deficiency of tissue existed, to perform the following operation:—Having carefully removed the diseased part by a V-shaped incision, he next divided the tissue remaining *horizontally*, carrying the cuts as far into the cheek on each side as he deemed necessary, after the manner of Horn; but in order to take off the strain from the flap, he made a *semilunar incision* in the cheek, just beyond the base of each. This enabled him to bring the parts together without difficulty; and the only objection to his operation is the danger of wounding the larger vessels, nerves, and ducts of the cheek, in making the semilunar divisions. This operation is spoken of by Galen, Paulus, and others, and was imitated by Guillemeau and Thèvenin, who made *straight* instead of *semilunar* incisions.

*Operation of M. Serres.*—It sometimes happens that the disease is confined to the integuments or subjacent muscles, leaving the mucous lining of the lip perfectly sound. In such cases, Serres *cuts away only the affected part*, and then *turns the mucous membrane over* the margin of what is subsequently to form the lip. A few stitches are sufficient to hold it in place; and union by the first intention usually occurring, a very natural and useful organ may thus be made. This method, however, will only answer in cases of *superficial* and *recent* disease,

*Operation of I. N. Roux.*—After removing the affected tissues, and forming suitable flaps of the adjacent parts, M Roux takes away with the saw or cutting instruments the *prominent centre of the maxillary bones*, so as to make room for the proper and easy adjustment of the integuments intended to replace the organ destroyed. I have never, as yet, met with any instance of a defect that required for its relief the performance of so severe an operation, and am not disposed to advise its employment, inasmuch as I believe *most*, if not *all*, cases may be cured with much less suffering and hazard by operations equally successful in their results. Cambrelin, Thomas, Nichet, and Velpeau, however, have all had recourse to it, and with, according to their reports, decided advantage.

*Operation of P. Roux.*—Professor Roux, not satisfied with the measures of his namesake, goes so far as to *saw out an inch or more* of the bone, and then by drawing the lateral flaps towards each other, he thus diminishes the *breadth* of that part of the face involved in the disease. Then detaching the flaps, he draws them across the opening in the bone, and the sutures which hold and unite the soft parts are, for the most part, sufficient to hold the bones in their proper places.

*Operation of Mr. Morgan.*—The operation of Mr. Morgan consists in, *first*, removing the entire lip by a *semilunar incision*, the *concavity* of which is uppermost; and *second*, in making an incision also curvilinear and parallel to, and about an inch or more *below* the first. The skin included between the two is then carefully detached, except at its extremities, and lifted into the place occupied by the diseased lip. Velpeau gives another explanation of this plan of Morgan; but from all I can ascertain, the process, as just described, was the one practised by that gentleman,

*Operation of Blasius.*—M. Blasius has performed a very simple operation, when the tumour was large; and according to his statement, with decided success. After removing the diseased mass by a common V-shaped incision, he next divided the integuments along the base of the lower jaw, by two incision which commenced at the entering angle of the V, and extended an inch or more in the direction specified. Lifting the flaps, he made them occupy the place of the original lips. It will be perceived that this plan is somewhat similar to the one employed in the case I have just reported.

*Operation of Dieffenbach.*—This extraordinary surgeon has, among many other plans for restoring the lip, performed one apparently hazardous and severe, but, nevertheless, according to the reports of others as well as those of Dieffenbach himself, exceedingly useful. The following description is taken from Zeis:

"Having pared away the useless remains of the former diseased lip, or separated the cicatrised margin, a horizontal incision, about two inches long, is carried from either angle of the mouth outwards, through the cheeks, so as to throw the mouth widely open. The length of these incisions must be regulated according to the width of the mouth; or, as a general rule, the combined incisions must somewhat exceed in length the breadth of the upper lip. From the outer point of each of these, another incision is next carried obliquely downwards and towards the median line; the section in this case likewise extending through the whole thickness of the cheek. Thus, by means of the first operation for paring the cicatrix, and by the succeeding horizontal and vertical incisions, a flap will be prepared on either side to replace the defective lip; this flap is of a quadrangular form, and maintains a connexion of more than one inch wide with the soft parts covering the tissue of the lower jaw. It may be useful further to separate the mucous membrane at its attachment to the gums, to allow of the more ready traction of the flaps."

The severe injury inflicted on the facial nerves, the large arteries and veins, and possibly the parotid duct, has rendered this operation anything but popular. Yet as already remarked, it has been performed with success by several, among whom is my colleague, Professor Pancoast.

*Operation of Liston.*—Any opinion of this truly great surgeon is always deserving of the utmost respect and attention, and, although my own observation leads me to a different conclusion in relation to the best mode of restoring a lip, I cannot for a moment hesitate to advise the repetition, whenever practicable, of his method, (a modification of the Indian,) by all who desire experience in this department of our art. It consists in first removing the diseased mass by a horizontal and two perpendicular cuts, or by one curvilinear in shape; and, second, in detaching a flap from the chin and neck, twisting it on its pedicle, placing it in the seat of the original lip, and there retaining it by suture. After adhesion has taken place, the pedicle is divided, and a "wedge-shaped" piece removed, so as to allow the flap to be laid down smoothly. This method, it is obvious, is frequently applied to the restoration of other parts, and will answer here exceedingly well in many cases; but I prefer the one I have reported, as there is *less scar*, and *less risk of sloughing* of the flaps. Mr. Liston proposed this operation ten years since, but some give the merit of the principle to Lallemand.

The operation reported by myself has been claimed by several, among whom are Dieffenbach, Blasius, Buchanan, and others. I can only say, that I performed it in 1834, and if any surgeon has a prior claim to the merit of its introduction into practice, I am both ready and willing to award to him all the honour that may accrue from its authorship.

## Figures and Tables

**Figure f1:**
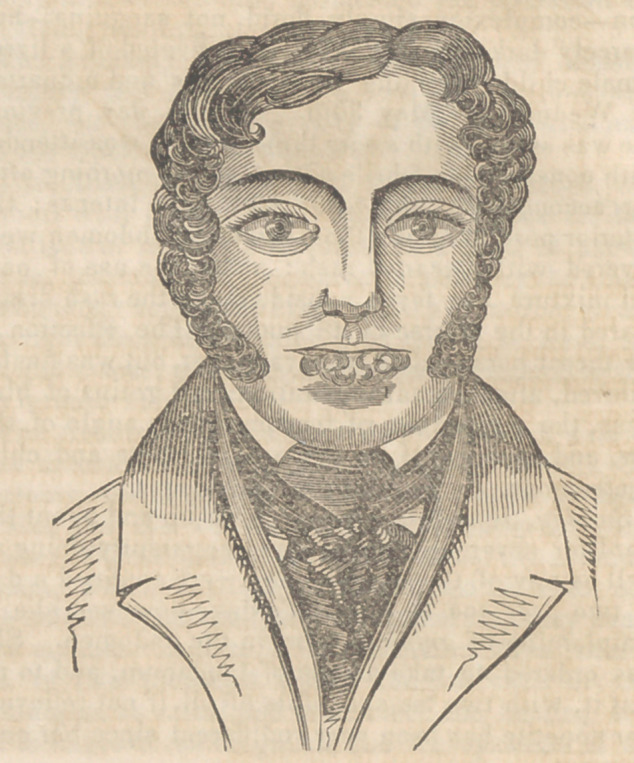


**Figure f2:**
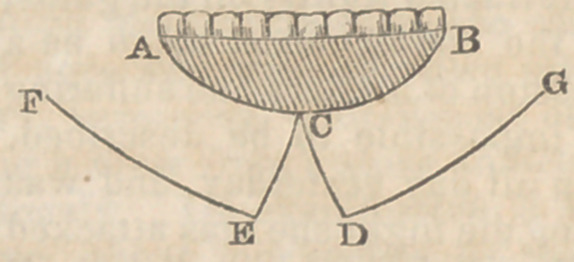


**Figure f3:**